# The effect of Mild Cognitive Impairment (MCI) on psychological distress among older adults in Ukraine

**DOI:** 10.1186/s12877-023-03906-1

**Published:** 2023-04-26

**Authors:** Jennifer Avery, David Thomas, Olha Myshakivska

**Affiliations:** 1 School of Nursing, Eastern Michigan University, Ypsilanti, MI USA; 2 School of Health Sciences, Eastern Michigan University, Ypsilanti, MI 48197 USA; 3 Taras Shevchenko National University, Kyiv, Ukraine

**Keywords:** Anxiety, Depression, Life stress paradigm, Mental health, Mild Cognitive Impairment (MCI)

## Abstract

**Objectives:**

Understanding the negative consequences of Mild Cognitive Impairment (MCI) in Ukraine among a population who have collectively experienced difficult life events, provided the impetus for the current study which analyzed whether the perception of psychological distress differed among older adults with two types of MCI (amnestic MCI [aMCI] & nonamnestic MCI [naMCI]) compared to their cognitively intact peers.

**Method:**

A sample of 132 older adults were selected from an outpatient regional hospital in Lviv, Ukraine and assigned into either an MCI or non-MCI control group. A demographic survey, and the Symptom Questionnaire (SQ) were administered to both groups.

**Results:**

Results of an ANOVA comparing the SQ sub-scales between the Ukrainian MCI and control groups were analyzed. A multiple hierarchical regression analysis assessed the predictive value of MoCA scores on the SQ sub-scales. Compared to adults in the MCI group, adults in the control group reported significantly lower rates of anxiety, somatic, depressive symptoms, and total psychological distress.

**Discussion:**

While the level of cognitive impairment was a significant predictor for each sub-type of distress, the explained variance was minimal suggesting that other factors also played a role. Reference was made to a similar MCI sample in the U.S. which had lower SQ psychological distress scores than the Ukraine sample, further suggesting possible environmental effects on symptoms. The importance of depression and anxiety screening and treatment for older adults with MCI was also discussed.

## Introduction

The on-going military conflict in eastern Ukraine has had a devastating consequence on the country’s population either directly (those living in the war-torn regions) or indirectly through displaced families and friends and the psychological distress associated with having a country at war. A plethora of research has documented the psychological harm affecting citizens of politically violent countries [1–4]. As in most cases of national upheaval, the most vulnerable of the population suffer the most. Older adults, who have not had an adequate social safety-net since the dissolution of the Soviet Union in 1991, are particularly affected in a country experiencing extreme social turmoil [5, 6]. Among older adults, those with cognitive impairments are even more at risk by the disruption of families as most people with mild cognitive impairment (MCI) and dementia in Ukraine need to rely on family or charitable organizations like the Red Cross for their care with formal networks and institutions either being inaccessible or prohibitively expensive. Even those who qualify for “free” government social care (i.e. living alone and not exceeding the minimum subsistence level defined by the government) still need to pay out of pocket care expenses for medications, supplies and special treatments. For the few, who can afford admittance to inpatient care, the availability of services can vary dramatically depending upon the local region’s allocation of funds and personal financial resources of the patient and/or family.

The psychological distress generated from political violence may have unanticipated outcomes for people with cognitive impairments who desperately need care within a system that is increasingly ill equipped to meet their needs. A body of research has supported the negative effects of psychological distress on normal cognition [7–11], and even more-so among people with a cognitively-compromised condition such as MCI and early dementia [12–14]. However, studying psychological distress among a sample of MCI patients enduring the consequences of a country at war, has not been evaluated. Better understanding the potential negative consequences of MCI in Ukraine among a population who have collectively experienced difficult life events, provided the impetus for the current study which aimed to analyze whether the perception of psychological distress differs among and between older adults with two MCI subtypes compared to their cognitively intact peers.

### Influences on psychological distress and MCI

#### MCI subtypes

Systematic reviews of psychological distress in persons with MCI have consistently highlighted the prevalence of depression and anxiety within the population [15, 16]. In addition, MCI can be broken down into the two subtypes of amnestic MCI (aMCI) with predominately deficits in memory and non-amnestic MCI (naMCI) with deficits largely outside of memory such as within executive functions, language, or visuospatial ability [17]. Diagnosis with either aMCI or naMCI is based on a person’s history, neuropsychological testing, and neuroimaging but remains somewhat subjective as typically more than one cognitive domain may be impacted. There is also conflicting evidence regarding the effects of psychological distress between the MCI subtypes. For example, while one study reported no significant differences in psychological distress by MCI subtype [18], others have demonstrated higher rates of depression in persons with aMCI compared to naMCI [19–21]. Psychological distress influenced by uncertainty and coping, is the final consequence of MCI, commonly identified through different reactions, among them anger, sadness, self-worth [22]. Studies conducted with samples in the U.S. demonstrated significant differences between the symptoms of sub-types of MCI with naMCI reporting higher degrees of distress than those with aMCI [23, 24].

#### Psychological hardiness

In Ukraine, distress may be interpreted differently by its older population compared to other societies due to a buffering effect created by high levels of psychological hardiness and resiliency. Numerous studies have reported the effect of life experiences on shaping the degree of hardiness, which includes having experienced many life changes, overcoming adversities and succeeding against the odds [25–28]. Older adults in Ukraine have experienced many adversities over the decades from Stalinism and famine, Soviet rule and more recently political strife, annexation and war. Perhaps Ukraine offers a unique perspective as to how psychological distress is experienced among both cognitively-intact and compromised older adults due to its historical and contemporary struggles.

#### Life stress paradigm

The Life Stress Paradigm [29] offers some guidance for understanding the possible relationships among living in a politically unstable country, cognition, and psychological distress. The paradigm demonstrates a negative relationship between stressors and resources while also supporting a positive relationship between stressors and distress, and negative relationship between resources and distress [29]. In other words, as stressors such as living in a politically unstable country increase, resources such as cognitive reserve decrease and psychological distress increases. This study focuses on evaluating part of the paradigm between cognition and psychological distress.

## Study aims

The following aims guided the study: (1) To compare levels of psychological distress between an MCI and a cognitively intact control group of older adults in Ukraine. (2) To compare differences in the levels of psychological distress between MCI subtypes (aMCI & naMCI). (3) To evaluate the potential impact of cognition on psychological distress.

## Methods

### Participants

A sample of participants were assigned from a pool of outpatient community members that receive health services through a network of primary-care physicians within the network of the regional psychiatric hospital in Lviv, Ukraine, into either an MCI group (n = 66) or non-MCI group (*n* = 66) based upon their pre-existing diagnoses of either aMCI, naMCI, or no diagnosis of MCI. Inclusion criteria for the two groups comprised being 55 and over, and the results of the Montreal Cognitive Assessment (MoCA) with scores of 19–25 indicating MCI. Potential participants were excluded from the study if dementia was evident, as evident by scores below 19, or if there was a diagnosis of MCI with a significant medical or psychiatric condition present. There was a total of 37 patients excluded from the study based on the inclusion/exclusion criteria. IRB approval was granted from the participating institution (UHSRC #911550–1). All subjects provided written consent to participate in the study. During the study, there was a great deal of instability caused by an active mobilization process of the male population and an influx of casualties among army and civilians. Typical complaints conveyed by the participants in the study included a lack of confidence in the future, frustration, anxiety, and hopelessness.

### Procedure

Data was collected using one time in-person interviews to minimize sample attrition and missing data. A cognitive assessment (MoCA) was conducted from a pool of outpatients to confirm eligibility and placement into either the MCI or control group. Once all participants were identified, a demographic survey, and the Symptom Questionnaire (SQ) were administered. Breaks were offered between questionnaires as needed to decrease potential response burden. A psychiatrist (MD, PhD) representing the Lviv Regional Hospital network in Ukraine performed the testing and selection process. All participants signed a conformed consent agreement approved by the institution’s Human-Subjects Research Program. Members of the research team, went through their respective IRB process which included subject confidentiality training.

### Assessment instruments

The Symptom Questionnaire (SQ) has been widely used since its initial publication to operationalize psychological distress in older adults [28]. Although the SQ has not been used widely in research involving older adults with MCI, the brevity and simplicity of the tool that includes dichotomous items (i.e. yes/no or true/false) would seem compatible with the defined sample. The SQ contained 92 dichotomous items of which 68 items indicate symptoms of psychological distress and 24 items and antonyms indication psychological well-being. Items are scored either 0–1 with 1 being indicative of psychological distress, so higher scores represent higher levels of distress. In addition to providing an overall psychological distress score, subscales on specific distress include depression, anxiety, anger-hostility, and somatic symptoms. Criterion-related validity was established with strong correlations with other existing instruments such as the Hopkins Symptom Checklist and the Hamilton Rating Scale for Depression. Reliability of the SQ was demonstrated by using test–retest reliability ranging from 0.57 to 0.95 using Cronbach’s alpha scores [30]. Translation of the Symptom Questionnaire (SQ) from English to Ukrainian was conducted by a certified translator. In addition, reverse translation of the questionnaire from Ukrainian back to English was conducted by a separate certified translator to help support the accuracy of the translation.

The Montreal Cognitive Assessment (MoCA) was originally developed as a brief screening tool for people with MCI [31] and has over the years become a well-established instrument used in assessing a variety of neurological cognitive impairments. The MoCA has been translated and validated and in multiple languages, including Ukrainian, in its assessing attention, memory, language, visual-spatial skills, concentration, orientation. and executive functions. The MoCA has a maximum score of 30 and can be administered in a relatively short time period. Lower scores on the MoCA are indicative of higher levels of cognitive impairment.

### Statistical analyses

Data analysis was performed using SPSS statistical software. All data was assessed for frequencies, mean, median, mode, and outliers. Differences in demographic data between samples (e.g. age, gender and education) were assessed using one-way analysis of variance (ANOVA) or *X*^2^ as appropriate. To address the aims of the study, an ANOVA was used to assess differences on the SQ sub-scales between the MCI and control groups, followed by the aMCI, naMCI and control groups. Finally, to assess the potential impact of cognitive impairment on psychological distress within the sample, multiple hierarchical regression analyses were used to control for age and years of education and to assess the predictive value of MoCA scores on the SQ subscales.

## Results

The age of the sample (*n* = 132) ranged from 54–86 years with a mean age of 65 years. Compared to adults in the MCI group, adults in the control group were significantly younger (*F*(1, 130) = 6.42, *p* = 0.01), reported more years of education (*F*(1, 129) = 4.78, *p* = 0.03), and as expected, scored higher on the MoCA (*F*(1, 130) = 124.15, *p* < 0.001), see Table 1 below. Between the MCI subtypes of aMCI (*n* = 39) vs. naMCI (*n* = 28), there were no significant differences in age (*F*(1, 64) = 0.13, *p* = 0.72), years of education (*F*(1, 64) = 2.31, *p* = 0.13), or scores on the MoCA (*F*(1, 64) = 2.85, *p* = 0.10). (Table 1).

**Table 1:** Subject demographics

	MCI Group (*n* = 66)	Control Group (*n* = 66)^a^
Age *Mean, SD* (Range)	66.77, 7.35 (54 – 83)	63.48, 7.56 (54 – 86)
Years of Education *Mean, SD* (Range)	13.74, 3.61 (6 – 22)	15.09, 3.45 (10 – 35)
MoCA Total *Mean, SD* (Range)	21.24, 3.41 (11 – 27)	26.70, 2.05 (21 – 30)
Gender *n* (%)		
Female	35 (53.0%)	46 (69.7%)
Male	22 (33.3%)	11 (16.7%)
Unspecified or Unclear	9 (13.6%)	9 (13.6%)

^a^*n* = 65 for Years of Education

Total scores for psychological distress ranged from 1–81 (max possible score = 92) with an average score of 27.80 (*SD* = 16.28) for the MCI group and 21.48 (*SD* = 12.32) for the control group. Compared to adults in the MCI group, adults in the control group reported significantly lower rates of anxiety symptoms (*F*(1, 130) = 9.61, *p* = 0.002), somatic symptoms (*F*(1, 130) = 8.38, *p* = 0.004), depressive symptoms (*F*(1, 130) = 4.40, *p* = 0.04), and total psychological distress (*F*(1, 130) = 6.32, *p* = 0.01). There was no significant differences in anger-hostility symptoms (*F*(1, 130) = 2.64, *p* = 0.11). The clinical importance of these differences is highlighted when considering the clinical range of each sub-scale (Table 2). Between the MCI subtypes (aMCI vs naMCI) there were no significant differences on any of the sub-scales or total psychological distress (*F*(1, 64) = 0.19–1.15, *p* = 0.29–0.66).

**Table 2:** Number of subjects within each clinical range

Subscale	Clinical Ranges	MCI Group *n* (%)	Control Group *n* (%)
Anxiety	Normal ≤ 7 Moderate = 8–11 Substantial to Severe ≥ 12*	51 (77.3%) 8 (12.1%) 7 (10.6%)	57 (86.4%) 9 (13.6%) 0 (0%)
Depression	Normal ≤ 6^*^ Moderate = 7–9 Substantial to Severe ≥ 10	47 (71.2%) 11 (16.7%) 8 (12.1%)	57 (86.4%) 5 (7.6%) 4 (6.1%)
Somatic Symptoms	Normal ≤ 8^*^ Moderate = 9–13^*^ Substantial to Severe ≥ 14	33 (50%) 29 (42.9%) 4 (6.1%)	53 (80.3%) 7 (10.6%) 6 (9.1%)
Anger-Hostility	Normal ≤ 7^*^ Moderate = 8–12 Substantial to Severe ≥ 13	58 (87.9%) 6 (9.1%) 2 (3%)	64 (97%) 2 (3%) 0 (0%)

^*^Denotes significant difference between MCI and Control groups at *p* < 0.05

To assess for the impact of cognitive impairment on psychological distress, multiple hierarchical regression analyses were conducted by controlling for age and years of education (Block 1) and assessing the predictive value of MoCA scores on the Symptom Questionnaire sub-scales. Model diagnostics supported the use of normal linear regression modeling. Each model significantly predicted some level of variance among the symptom sub-scales (Table 3), highlighting the relationship between cognitive impairment and psychological distress**.** However, the relationship is rather small. Specifically, after accounting for age and education, level of cognitive impairment (scores on the MoCA) significantly accounts for 9% of the variance in Anxiety, 8% of the variance in Anger-Hostility, and 4% of the variance in Depression and Somatic symptoms.

**Table 3:** Testing of relationships between cognitive impairment and psychological distress (*n* = 131)

	Anxiety		Depression		Anger-Hostility		Somatic	
Variables	*B* (*SE*)	β	*B* (*SE*)	β	*B* (*SE*)	β	*B* (*SE*)	β
Block 1: Control								
(Constant)	3.14 (3.20)		-0.39 (2.99)		3.57 (2.72)		0.90 (3.82)	
Age	0.07 (0.04)	0.14	0.10 (0.04)	0.23**	0.01 (0.04)	0.01	0.17 (0.05)	0.28***
Education	-0.23 (0.09)	-0.23**	-0.15 (0.08)	-0.16	-0.07 (0.08)	-0.09	-0.36 (0.11)	-0.28**
*R*^*2*^ Change	0.08		0.09		0.01		0.19	
*F* Ratio for *R*^*2*^ Change	5.87**		6.56**		0.53		14.62***	
Block 2: Cognitive Impairment								
(Constant)	11.90 (3.90)		5.26 (3.74)		10.47 (3.39)		7.98 (4.78)	
Age	0.02 (0.04)	0.05	0.08 (0.04)	0.17	-0.03 (0.04)	-0.08	0.13 (0.05)	0.22**
Education	-0.15 (0.09)	-0.15	-0.10 (0.08)	-0.10	-0.01 (0.07)	-0.01	-0.29 (0.11)	-0.23**
MoCA	-0.30 (0.08)	-0.32***	-0.19 (0.08)	0.22**	-0.24 (0.07)	-0.31***	-0.24 (0.10)	-0.20*
*R*^*2*^ Change	0.09		0.04		0.08		0.04	
*F* Ratio for *R*^*2*^ Change	13.09***		5.92**		11.44***		5.69*	
*R*^*2*^(Adjusted *R*)	0.17 (0.15)		0.13 (0.04)		0.09 (0.07)		0.22 (0.20)	

*B (SE)* Unstandardized Coefficients, *β* Standardized Coefficient

^*^p ≤ 0.05

^**^p ≤ 0.01

^***^p ≤ 0.001

## Discussion

In Ukraine, an ever changing political, ecological and social environment demands constant emotional adaptation. Older adults with MCI are especially vulnerable to these changes. Living in an under-resourced country that is experiencing extreme external stressors caused by political strife only adds to the vulnerability of an already at-risk population. The findings of this study support prior research that suggested adults with cognitive impairments have greater difficulty compared to a reference of non-impaired adults, in coping with stressors manifested in a broad array of negative symptoms. It was notable that there were significant differences between the MCI and Control group in age and educational level. It has been reported that lower education has correlated with an increase in MCI development. Petersen [17] reported that fluid cognitive ability (FCA) may better equip the higher educated person to deal with stress. Stern [32] discussed the role of increased education and its positive effect on cognitive resiliency allowing the person experiencing cognitive decline to better cope with life’s demands. Whereas, age has not been correlated with perceived stressfulness of a situation or one’s coping efficacy [33].

While the level of cognitive impairment was a significant predictor for each sub-type of distress, the amount of explained variance was minimal. This suggests the presence of other factors that may have contributed to distress, such as living in an unstable and life-threatening environment consistent with the Life Stress Paradigm. Of particular concern in this study was the clinical implications of differences on the anxiety and depression sub-scales. Significantly more participants were in the moderate and severe ranges of anxiety and depression among the MCI group compared to the control. This finding mirrors previous research from a recent systematic review and meta-analysis which concluded that older adults with MCI are likely to exhibit higher than normal levels of depression [34]. Interestingly, a similar study among a U.S. MCI sample (20) reported significantly lower rates of anxiety symptoms (*F*(1, 155) = 7.04, *p* = 0.009), somatic symptoms (*F*(1, 155) = 67.05, *p* < 0.001), depressive symptoms (*F*(1, 155) = 13.42, *p* < 0.001), and total psychological distress (*F*(1, 155) = 23.45, *p* < 0.001) compared to the Ukrainian MCI group from this study. A similar cross cultural comparison in a more controlled study might reveal that certain environmental conditions help explain the variance noted in this study, with extreme societal turmoil potentially playing a primary role regardless of any psychological hardiness traits the Ukrainian sample may possess. There remains a dearth of research comparing societies experiencing life threatening events, such as political unrest, to those not experiencing such events, among older adults with MCI. Further investigation comparing environmental influences on psychological distress among people with MCI is warranted. The results of the study also emphasize**s** the importance of psychological screening and treatment for older adults with MCI, who may not otherwise report symptoms. The findings revealed that there were no significant differences between the aMCI and naMCI groups, suggesting that the need for screening of psychological distress should not differ by MCI subtype. The importance of early detection of cognitive and psychological impairment among its older population was recently realized in another former Soviet republic country, Kazakhstan, where the first Memory Center opened in 2015 with the mission of providing screening and treatment services for older adults in the Almaty region [35]. The limited availability of medical care for cognitively impaired older adults in similarly under-resourced countries underscores the significance of early detection to increase the viability of less intrusive, early intervention options.

The global increase in population aging accentuates the need to recognize common challenges that exist across nations in maintaining independence and quality-of-life among its older adults with cognitive decline. Viewing MCI as only a cognitive impairment without understanding the added vulnerability to emotional and psychological problems overlooks a critical component to health and wellbeing. The present study was an attempt to better understand how people with MCI in Ukraine may interpret psychological distress differently from their non-impaired peers.

Limitations of the study includes the use of unmatched groups in age and education between the MCI and reference group, although attempts were made to statistically control these differences. Also, the use of a translated version of the Symptom Questionnaire could be considered a limitation. Although a certified translator was used, the instrument was never validated with a Ukrainian sample. Furthermore, the MoCA may not have been sensitive to the variability of the Symptom Questionnaire. Additional cognitive measures should be explored with better precision in noting subtleties of the Symptom Questionnaire. Additional research is needed to compare the impact of living in a politically unstable environment on psychological distress to more stable societies.

## Data Availability

The datasets used and/or analyzed are available from JA (javery10@emich.edu) upon request.

## References

[1] Wildt H, Umanos J, Khanzada NK, Saleh M, Rahman HU, Zakowski SG (2017). War trauma, psychological distress, and coping among Afghan civilians seeking primary health care. Int Persp in Psychol Res Pract Consult.

[2] Hobfoll S, Hall B, Canetti D (2012). Political violence, psychological distress, and perceived health: A longitudinal investigation in the Palestinian Authority. Psychol Trauma Theor Res Pract Polic.

[3] Rasmussen A, Nguyen L, Wilkinson J, Vundla S, Raghavan S, Miller KE, Keller AS (2010). Rates and impact of trauma and current stressors among Darfuri refugees in Eastern Chad. Am J of Orthol.

[4] Morina N, von Collani G (2006). Impact of war-related traumatic events on self-evaluation and subjective wellbeing. Traumatology.

[5] Russell D, Cutrona C (1991). Social support and depressive symptoms among the elderly: Test of a process model. Psychol Aging.

[6] Cutron C, Russell D, Rose J (1986). Social support and adaptation to stress by the elderly. Psychol Aging.

[7] Neupert S, Almeida D, Mroczek D, Spiro A (2006). Daily stressors and memory failures in a naturalistic setting: findings from the VA normative aging study. Psychol Aging.

[8] Paradise MB, Glozier NS, Naismith SL, Davenport TA, Hickie IB (2011). Subjective memory complaints, vascular risk factors and psychological distress in the middle-aged: a cross sectional study. BMC Psychol.

[9] Peavy G, Salmon D, Jacobson M, Hervey A, Gamst A, Wolfson T (2009). Effects of chronic stress on memory decline in cognitively normal and mildly impaired older adults. Am J of Psychol.

[10] Rosnick C. Stress and cognitive performance in older adults [dissertation on the Internet]. Tampa (USA): University of South Florida; 2005. [cited 2023 Apr 25]. Available from: https://digitalcommons.usf.edu/cgi/viewcontent.cgi?article=1836&context=etd.

[11] Stawski R, Almeida D, Lachlan M, Tun P, Rosnick C (2010). Fluid cognitive ability is associated with greater exposure and smaller reactions to daily stressors. Psychol Aging.

[12] Devier D, Pelton G, Tabert M, Liu X, Cuasay K, Eisenstadt R (2009). The impact of anxiety on conversion from mild cognitive impairment to Alzheimer`s disease. Int J of Geriat Psychol.

[13] McIlvane J, Popa M, Robinson B, Houseweart K, Haley W (2008). Perceptions of illness, coping, and well-being in persons with mild cognitive impairment and their care partners. Alzheimer Dis Assoc Disord.

[14] Rickenbach EH, Condeelis KL, Haley WE (2015). Daily stressors and emotional reactivity in individuals with mild cognitive impairment and cognitively health controls. Psychol and Aging.

[15] Apostolova LG, Cummings JL (2008). Neuropsychiatric manifestations in mild cognitive impairment: a systematic review of the literature. Dem Geri Cog Disord.

[16] Monastero R, Mangialasche F, Camarda C, Ercolani S, Camarda R (2009). A systematic review of neuropsychiatric symptoms in mild cognitive impairment. J of Alzheimer Dis.

[17] Petersen RC, Caracciolo B, Brayne C, Gauthier S, Jelic V, Fratiglioni L (2014). Mild cognitive impairment: a concept in evolution. J of Intern Med.

[18] Lee K, Cho H, Hong C, Kim D, Oh B (2008). Differences in neuropsychiatric symptoms according to mild cognitive impairment subtypes in the community. Dement and Geriatr Cogn Disord.

[19] Edwards ER, Spira AP, Barnes DE, Yaffe K (2009). Neuropsychiatric symptoms in mild cognitive impairment: Differences by subtype and progression to dementia. Inter J of Geriatr Psychol.

[20] Rozzini L, ViciniChilovi B, Conti M, Delrio I, Borroni B, Trabucchi M, Padovani A (2008). Neuropsyhiatric symptoms in amnestic and nonamnestic mild cognitive impairment. Dement and Geriatr Cogn Disord.

[21] Shahnawaz Z, Reppermund S, Brodaty H, Crawford JD, Draper B, Trollor JN, Sachdev PS (2013). Prevalence and characteristics of depression in mild cognitive impairment: The Sydney Memory and Ageing Study. Acta Psychol Scand.

[22] Avery JS. Relationships among uncertainty, coping, and psychological distress in older adults with mild cognitive impairment [dissertation on the Internet]. Milwaukee (USA): Marquette University; 2014. [cited 2023 Apr 25]. Available from https://epublications.marquette.edu/dissertations_mu/407/.

[23] Rosenberg P, Mielke M, Appleby B, Oh E, Lyketsos J-M, Lyketosos C (2011). Neuropsychiatric symptoms in MCI subtypes: The importance of executive dysfunction. Int J Geriatr Psychol.

[24] Beasley M, Thompson T, Davidson J (2003). Resilience in responses to life stress: The effects of coping style and cognitive hardiness. Pers Individ Dif.

[25] Couto M, Koller S, Novo R (2011). Stressful life events and psychological well-being in a Brazilian sample of older persons: the role of resilience. Ageing Int.

[26] Hardy S, Concato J, Gill T (2002). Stressful life events among community-living older persons. J Gen Intern Med.

[27] Robertson I, Cooper G (2013). Resilience. Stress Health J Int Soc Invest Stress.

[28] Webb CM. Recollected early life experiences and hardiness [dissertation]. Chicago (USA): The University of Chicago; 1994.

[29] Easel WM, Lin N (1991). The life stress paradigm and psychological distress. J of Health and Soc Behav.

[30] Kellner R (1987). A symptom questionnaire. J of Clin Psychol.

[31] Nasreddine Z, Phillips N, Bedirian V, Charbonneau S, Whitehall V, Collin I, Chertkow H (2005). The Montreal Cognitive Assessment, MoCA: a brief screening tool for mild cognitive impairment. J Am Geriatr Socie.

[32] Stern Y (2012). Cognitive reserve and ageing and Alzheimer’s disease. Lancet Neurol.

[33] Aldwin C, Sutton K, Chiara G, Spiro A (1996). Age differences in stress, coping, and appraisal: Finding from the Normative Aging Study. J Gerotol Psychol Sci.

[34] Ismail Z, Elbayoumi H, Fischer CE, Hogan DB, Milikin CB, Schweizer T (2017). Prevalence of depression in patients with mild cognitive impairment: a systematic reviewand meta-analysis. JAMA Psychol.

[35] Thomas DW, Yeshmanova A, Akanova A, Umutbayeva G, Abikulova A, Chaikovska V (2016). Development of a memory center for older adults in Almaty, Kazakhstan. Dem The Int J Soc Res Pract.

